# High-performance asymmetric optical transmission based on coupled complementary subwavelength gratings

**DOI:** 10.1038/s41598-019-53586-4

**Published:** 2019-11-19

**Authors:** Shuang Li, Li-rong Huang, Yong-hong Ling, Wen-bing Liu, Chun-fa Ba, Han-hui Li

**Affiliations:** 1 Wuhan National Laboratory for Optoelectronics, Huazhong University of Science and Technology, 1037 Luoyu Rd, Wuhan, 430074 China; 2 Wuhan Maritime Communication Research Institute, Hubei, 430079 China

**Keywords:** Nanophotonics and plasmonics, Optical physics

## Abstract

Asymmetric transmission (AT) devices are fundamental elements for optical computing and information processing. We here propose an AT device consisting of a pair of coupled complementary subwavelength gratings. Different from previous works, asymmetric dielectric environment is employed for unidirectional excitation of surface plasmon polaritons (SPPs) and thus asymmetric optical transmission, and near-field coupling effect inherent in the coupled complementary structure is exploited to enhance forward transmission and AT behavior, and determine operation bandwidth as well. The influence of asymmetric dielectric environment, effect of vertical and lateral couplings, interactions of electric- and magnetic-dipole moments and the realization of Kerker conditions, are investigated in depth to unearth the AT mechanism and performance. High-performance AT with large forward transmittance of 0.96 and broad bandwidth of 174 nm is achieved at wavelength 1250 nm. Our work helps people to gain a better understanding of near-filed coupling effect in coupled complementary structures, expand their application fields, and it also offers an alternate way to high-performance AT devices.

## Introduction

Asymmetric optical transmission devices, exhibiting different transmittances for opposite propagation directions, have attracted enormous research interests because they play a fundamental role in optical signal processing^1,2^, noise control and cancelation^3^, optical diodes^4,5^, optical interconnection and multiplexing^6–8^, optical systems for one side detection/sensing^9,10^, and so on. Traditional approaches for asymmetric transmission (AT), such as using magneto-optic medium^11^ and nonlinear medium^4^, have been extensively demonstrated. In recent years, artificial structures have also been reported for realizing AT, including photonic crystals^12^, metamaterials^13–15^, subwavelength metallic or dielectric gratings^16,17^, etc.

Surface plasmon polaritons (SPPs) have been shown to exhibit strong enhancement of local field due to the strong confinement of light to the metallic surface^18,19^. Recently, AT effects based on unidirectional excitation of SPPs have also been studied by utilizing double gratings with different periods^20–22^, multilayer metasurfaces^23^, and asymmetric metallic gratings with one or multiple subwavelength slits^24,25^. However, these structures usually suffer from larger insertion loss because SPPs need to tunnel through metallic thin film or transmit through the subwavelength slit(s) of the metallic structures, which causes optical loss and sacrifices transmittance to some degree, thus limiting their applications.

In this paper, we propose an AT device based on coupled complementary subwavelength gratings. According to Babinet’s law, coupled complementary bilayer structures naturally possess electric and effective magnetic responses over a wide range of electromagnetic spectrum^26,27^. The strong coupling effects inherently existing in the bilayer structures can provide strong light-matter interactions and multi-dimensional control on electromagnetic waves, therefore having significant advantages in improving the performances of nanophotonic devices^28^. For example, coupled complementary structures have been employed to realize wide-angle near-infrared polarizer with extremely high extinction ratio^29^, enhance visible light transmission^30^, and improve the coloration resolution of metasurface-based plastic consumer products^31^, etc. Here, we use coupled complementary subwavelength gratings to construct a high-performance AT device, in which asymmetric dielectric environment is utilized to unidirectionally excite SPPs, and near-field coupling inherently existing between the upper and lower gratings enables electric- and magnetic-dipole moments to interact with each other, thus significantly enhancing forward transmittance, asymmetric transmission and operation bandwidth.

We organize our paper as follows. First, we present the structure and operation principle of the AT device; then, we give the results and discussion. Finally, a brief discussion is made.

## Device Structure and Operation Principle

### Device structure

**Figure 1:**
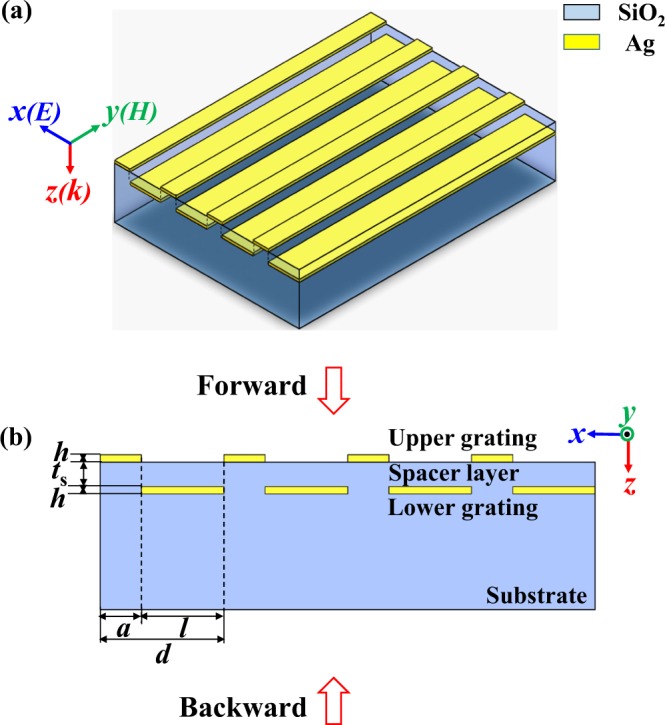
Device structure. (**a**) Schematic diagram and (**b**) cross-section view of the AT device. Parameters are *a* = 300 nm, *l* = 600 nm, *d* = 900 nm, *h* = 50 nm and *t*_s_ = 200 nm.

Figure 1(a,b) depict the schematic diagram and cross-section view of the designed asymmetric transmission (AT) device, which consists of an upper silver (Ag) grating and a lower Ag grating embedded in a silica (SiO_2_) substrate. The two gratings have the same groove depth *h* = 50 nm and the same period *d* = 900 nm (smaller than operation wavelength). In each structural unit, the Ag strip in the upper grating has a width of *a* = 300 nm, while the Ag strip in the lower Ag grating, which is located just below the groove of the upper Ag grating, has a width of *l* = 600 nm. That is, the sum width of the two Ag strips is exactly equal to the period 900 nm, i.e., *d* = *a* + *l*, therefore the two gratings are geometrically complementary to each other. As the SiO_2_ dielectric spacer layer is very thin with a thickness *t*_s_ = 200 nm, strong near-field coupling effect exists between the upper and lower gratings^27,30,32^.

Compared with previously reported nonsymmetrical bilayer grating-based AT devices, ours has two distinct structural features.

First, in previous reports, the upper and lower gratings have different periods such that surface plasmon polaritons (SPPs) are unidirectionally excited^20–25^. In contrast, here the upper and lower gratings have the same period *d* = 900 nm, and asymmetric dielectric environment is used to realize the unidirectional excitation of SPPs. As depicted in Fig. 1(b), the lower grating is embedded in SiO_2_ dielectric environment, while the upper grating is surrounded with air/SiO_2_ environment.

The second distinct feature of our AT device is that, the bilayer gratings are of coupled complementary structure. It is well known that near-field coupling effect inherently exists in such structures^27,30^. As demonstrated later, this near-field coupling effect, through the coherence interference of electric- and magnetic-dipole moments and Kerker conditions, can greatly enhance forward transmittance at operation wavelength, increase AT effect, and also determine operation bandwidth.

### Operation principle

#### Asymmetric dielectric environment for asymmetric excitation of SPPs

To begin with, we explain how asymmetric dielectric environment can realize asymmetric excitation of SPPs (i.e., unidirectional SPPs excitation). The dispersion relation of SPPs can be written as^19^:

1 $${k}_{spp}={k}_{0}{|\frac{{\varepsilon }_{m}{\varepsilon }_{d}}{{\varepsilon }_{m}+{\varepsilon }_{d}}|}^{\frac{1}{2}}$$

where *k*_0_ is the wave vector of light in free space, *ε*_m_ and *ε*_d_ are the frequency-dependent permittivities of the metal and the dielectric material, respectively. According to Eq. (1), SPPs have larger wave vector than light in free space. In order to excite SPPs, the mismatch of wave vector should be compensated by introducing an extra wave vector. Furthermore, the wavelength of SPPs (i.e., *λ*_*spp*_ = 2π/*k*_*spp*_) is also dependent on the permittivities of surrounding media, hence the asymmetric dielectric environment enables the upper and lower gratings to stimulate SPPs with different wavelengths even if they have the same period *d*. Therefore, by carefully designing structural parameters, the proposed AT device is able to excite SPPs only for forward incident light, while not for backward incident light at the same frequency, thus realizing one-way SPPs excitation and thereby supporting asymmetric transmission.

#### Interactions of electric- and magnetic-dipole moments and Kerker conditions

Apart from having the ability to asymmetrically excite SPPs, our proposed coupled complementary structure also inherently possesses strong near-field coupling effect between the upper and lower gratings because the dielectric spacer layer is of subwavelength thickness. Here, Kerker conditions are adopted to illustrate electric and magnetic dipole coupling on light manipulation. Kerker conditions clarify the requirement for zero forward/backward scattering when dealing with electric and magnetic dipole responses^33^. This kind of unidirectional scattering can be realized by various micro/nano structures, including Huygens’ metasurfaces, high-dielectric nanoparticles or coupled metallic nanoparticles. Based on Kerker conditions, one can design high-performance asymmetric transmission devices, transmission arrays^34^, reflectors, polarization converters and frequency selective absorbers^35^. Now we explain how Kerker conditions are satisfied in our AT device.

When an *x*-polarized wave normally illuminates the AT device along the positive *z*-axis (i.e, the forward propagation direction), the light first hits the upper grating and excites electric-dipole moment ***p***_**x**_ along the *x*-axis, and according to the Babinet’s law^27,36–38^, the lower grating will be induced an antiparallel current to form a magnetic-dipole moment ***m***_**y**_ along the *y*-direction. Then the normalized forward/backward (i.e., along the positive or negative *z*-axis) scattering cross section can be expressed as^34,35,39^:

2 $$Q=\frac{{k}^{4}}{4\pi {\varepsilon }^{2}A{|{E}_{inc}|}^{2}}{|{p}_{x}\pm \frac{\sqrt{{\varepsilon }_{r}}{m}_{y}}{c}|}^{2}$$

where *k* is the wavenumber in a background material with electric permittivity *ɛ* = *ɛ*_0_*ɛ*_r_, *c* is the speed of light in free space, and |*E*_inc_| is the amplitude of the incident electric field. *A* is the geometrical cross section, and ± represents forward and backward scattering cross sections, respectively.

According to Eq. (2), it is possible that at a certain wavelength, electric- and magnetic-dipole moments are in phase, which satisfies the first Kerker condition, then their radiated light waves undergo constructive interference, and zero backward scattering occurs^35^.

3 $${p}_{x}-\frac{\sqrt{{\varepsilon }_{r}}{m}_{y}}{c}=0$$

In this case, the device can achieve high transmittance.

It is also possible that at other wavelengths, electric- and magnetic-dipole moments are out of phase by π, which satisfies the second Kerker condition, then their radiated light waves undergo destructive interference, and zero forward scattering takes place^35^.

4 $${p}_{x}+\frac{\sqrt{{\varepsilon }_{r}}{m}_{y}}{c}=0$$

In this case, the incoming light is mostly reflected, and transmittance has a minimal value.

While at other wavelengths, the incident *x*-polarized light neither satisfies the first nor the second Kerker condition, then it will be partly transmitted and partly reflected.

Therefore, with the assistance of unidirectional SPPs excitation and coherent interference of electric- and magnetic-dipole moments, the optimally-designed coupled complementary AT device can achieve high forward transmittance at the operation wavelength.

While for the backward propagation direction (i.e., the negative *z*-axis), most light is reflected by the lower grating because it has a large filling factor (*f*_2_ = *l*/*d* = 0.67)^20^. What’s more, as mentioned above, SPPs can’t be excited in this case, hence backward transmittance will be very low without the assistance of SPPs. As we know, a high forward transmittance and a low backward transmittance will naturally allow for a high-contrast ratio (i.e. the ratio of forward transmittance to backward transmittance).

## Results and Discussion

### Effect of asymmetric dielectric environment

**Figure 2:**
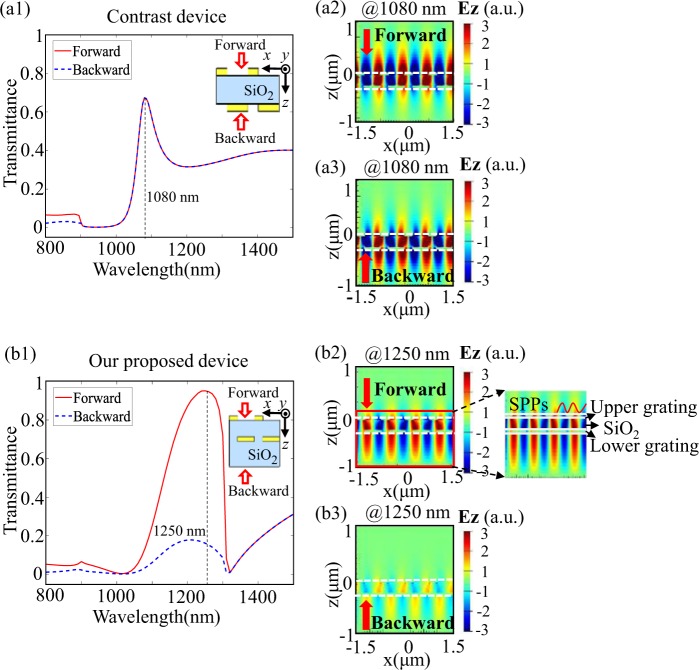
(**a1**) Forward and backward transmittance spectra of the contrast device in symmetric dielectric environment and its *E*_z_ electric field distributions at wavelength 1080 nm under (**a2**) forward and (**a3**) backward illumination. (**b1**) Forward and backward transmittance spectra of our proposed device in asymmetric dielectric environment and its *E*_z_ electric field distributions at wavelength 1250 nm under (**b2**) forward and (**b3**) backward illumination. A zoomed plot is given for the red solid region in (**b2**), and the dashed white rectangles represent the locations of the upper and lower gratings.

To begin with, we discuss the influence of asymmetric dielectric environment by comparing the AT performances of a contrast device and our proposed device. Figure 2(a1) shows the forward and backward transmission spectra of the contrast device, in which the upper and lower gratings are in symmetric dielectric environment and both face air/SiO_2_ interface (as shown in the inset of Fig. 2(a1)), while other parameters are kept the same as those of our proposed AT device. For example, the upper and lower gratings in both devices share the same period *d* = 900 nm, and the filling factors of the upper gratings in both cases are *f*_*1*_ = *a/d* = 0.33, while those of the lower gratings are both *f*_*2*_ = *l/d* = 0.67.

Figure 2(a2,a3) depict the *E*_z_ electric field distributions at the peak transmission wavelength of 1080 nm. As can be seen, both forward and backward light stimulate SPPs at the air/grating interface, and their transmittance spectra coincide with each other, indicating that no AT phenomenon takes place in the contrast device, even though its upper and lower gratings have different filling factors.

Figure 2(b1) shows the forward/backward transmittance spectra of our proposed device, in which the lower grating is completely buried in the SiO_2_ medium while the upper grating is located in air/SiO_2_ interface (as shown in the inset of Fig. 2(b1)), so the two gratings are in asymmetric dielectric environment. Figure 2(b2,b3) show the *E*_z_ electric field distributions at the peak transmission wavelength of 1250 nm, SPPs can be excited in the upper air/metal interface and decoupled to free space under forward illumination. While at the same incident wavelength under backward illumination, few SPPs are generated and most incoming light is reflected. In this case, forward propagating light gets a much higher transmittance than the backward propagating one.

It can be concluded that, the upper and lower gratings in symmetric dielectric environment will symmetrically excite SPPs, consequently resulting in symmetric optical transmission. In sharp contrast, our proposed device in asymmetric dielectric environment can promote the asymmetric excitation of SPPs, thereby leading to asymmetric optical transmission. In addition, it is interesting and important to note that, although the upper and lower gratings have different filling factors, it makes no contribution to asymmetric SPPs excitation and asymmetric optical transmission. For our coupled complementary structure device, which naturally has the same period for the upper and lower gratings, it is not the different filling factors but the asymmetric dielectric environment that results in asymmetric SPPs excitation and asymmetric optical transmission.

### Impacts of near-field coupling effect

Since our proposed AT device is composed of bilayer complementary gratings and the thickness of dielectric spacer layer is far less than operation wavelength, strong near-field coupling effect exists between the two gratings. It can be classified into vertical and lateral couplings, the first one is mainly determined by the spacer layer thickness *t*_s_, while the second one not only depends on the lateral misalignment *s* between the two gratings, but also indirectly relies on the spacer layer thickness *t*_s_.

### Vertical coupling

When the spacer layer thickness *t*_s_ is on the order of sub-wavelength, strong near-field coupling effect exists between the upper and lower gratings. In contrast, if the upper grating is far enough from the lower one, no coupling effect exists between them, then the total transmittance of the AT device equals to the product of the three transmittances, that is, *T*_total_ = *T*_1_ × *T*_2_ × *T*_3_, where *T*_total_ is the total transmittance, and *T*_1_, *T*_2_, *T*_3_ are the transmittances when the light passes through the individual upper grating, the individual spacer layer, and the individual lower grating, respectively.

**Figure 3:**
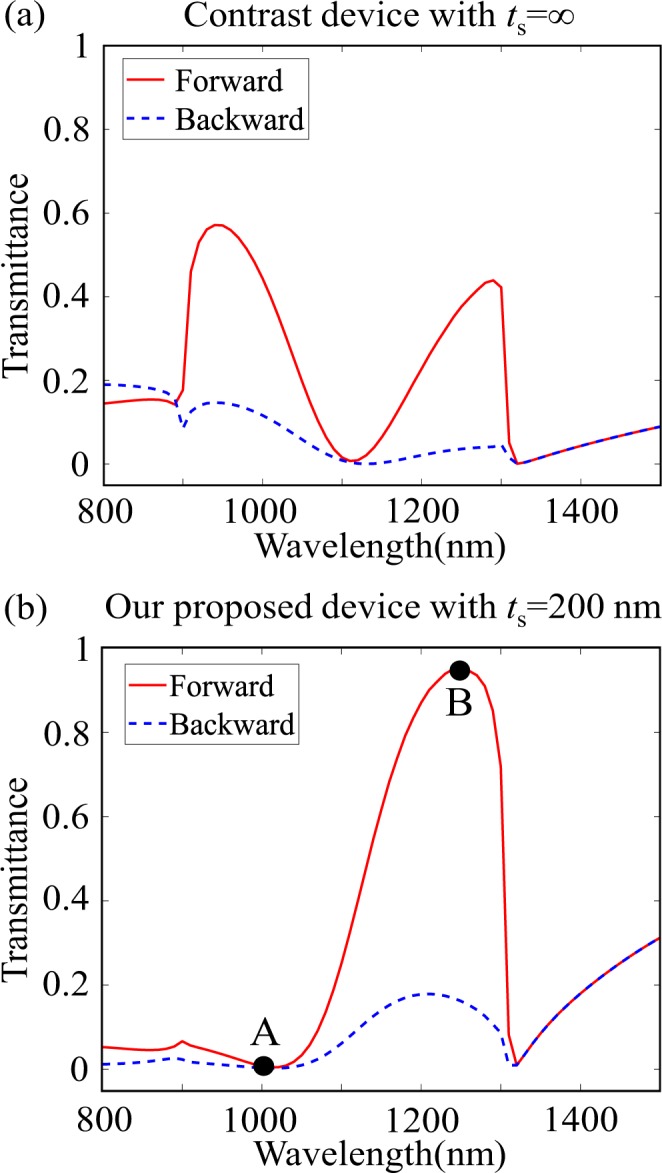
Transmittance spectra under forward and backward illumination for (**a**) the contrast device with *t*_s_ = **∞** and (**b**) our proposed device with *t*_s_ = 200 nm. Points “A” and “B” mark the two typical wavelengths discussed later.

We here study the effect of vertical coupling by comparing our proposed AT device with a contrast device which has *t*_s_ = ∞. Figure 3(a) shows the forward and backward transmittance spectra of the contrast device, which displays a certain degree of AT behavior originating from asymmetric dielectric environment. Although the forward and backward incident waves exhibit different transmittance, the contrast ratio is relatively lower.

However, when the upper and the lower gratings are close enough, for example, *t*_s_ is only 200 nm in our proposed AT device, strong near-field coupling effect takes place. The corresponding forward and backward transmittance spectra are shown in Fig. 3(b). Comparing with the contrast device without coupling effect shown in Fig. 3(a), our proposed device exhibits a greatly suppressed forward transmittance in 800~1050 nm waveband and significantly enhanced forward transmittance in 1100~1300 nm range. The peak forward transmittance is up to 0.96 at *λ* = 1250 nm, and the 3-dB bandwidth (It is defined as the wavelengths where the transmittance drops to half of the peak value.) is 174 nm (from 1130 nm to 1304 nm); while the backward transmittance is less than 0.16 within this wavelength range, indicating that our proposed device can carry out high-performance AT in a wide spectral range.

**Figure 4:**
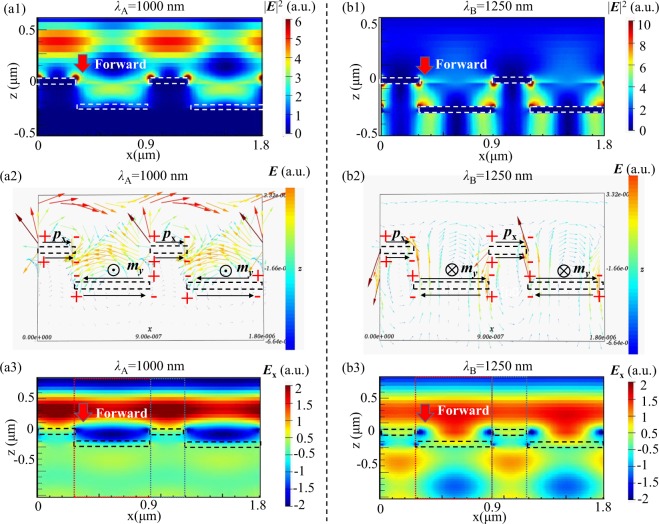
For our proposed device under forward illumination at *λ*_A_ = 1000 nm, (**a1**) electric intensity |*E*|^2^ patterns, (**a2**) electric field vector distribution and (**a3**) *E*_x_ electric field distribution in the *x-z* plane. At *λ*_B_ = 1250 nm, (**b1**) electric intensity |*E*|^2^ patterns, (**b2**) electric field vector distribution and (**b3**) *E*_x_ electric field distribution in the *x-z* plane. The dotted rectangles represent the locations of the upper and lower gratings. “+” and “−” in (**a2,b2**) refer to the signs of charge distribution.

To further investigate the impact of vertical coupling, we choose two typical wavelengths, one is *λ*_A_ = 1000 nm at which forward transmittance has a minimal value of 0.02, and the other is *λ*_B_ = 1250 nm at which the forward transmittance reaches the maximal value of 0.96. Figure 4(a1–a3) and Fig. 4(b1–b3) plot their electric field distributions in the *x-z* plane under forward incidence.

As shown in Fig. 4(a1) which plots the electric intensity |*E*|_2_ at *λ*_A_ = 1000 nm, the incident light undergoes strong reflection. As can be seen from the corrseponding electric field vector distribution in Fig. 4(a2) (the direction of the arrows represents the flow of current), when illuminated by *x*-polarized light, the upper grating stimulates parallel current to form electric-dipole moment ***p***_**x**_ oscillating in the *x*-direction; while in the lower grating, anti-parallel current forms magnetic-dipole moment ***m***_**y**_ along the *y*-direction. Furthermore, from the *E*_x_ electric field distribution in Fig. 4(a3), one can observe that *E*_x_ in the upper grating has positive sign (represented by red color in the color map) while that in the lower grating has negative value (indicated by blue color in the color map), signifying that the radiated wave from ***p***_**x**_ and that from ***m***_**y**_ are nearly out of phase by π, thereby they will interfere destructively in the exit medium^40^. Consequently, the second Kerker condition is met, zero forward scattering occurs, and very little electromagnetic energy can be transmitted through the device. As a result, the forward transmission is as low as 0.02, while the reflectivity is nearly 0.95 at *λ*_A_ = 1000 nm.

Figure 4(b1) depicts the electric intensity |*E*|_2_ at *λ*_B_ = 1250 nm, the incident light expericences strong transmission. As can be seen from the corresponding electric field vector distribution in Fig. 4(b2) (the direction of the arrows represents the flow of current), when illuminated by *x*-polarized light, the upper grating excites parallel current to form electric-dipole moment ***p***_**x**_ oscillating in the *x*-direction; while anti-parallel current induced in the lower grating produces magnetic-dipole moment ***m***_**y**_ along the *y*-direction. Furthermore, from the *E*_x_ electric field distribution in Fig. 4(b3), one can find that *E*_x_ in the upper grating has positive value (represented by yellow color in the color map) while that in the lower grating also has positive value (also shown by yellow color in the color map), indicating that the radiated wave from ***p***_**x**_ and that from ***m***_**y**_ are nearly in phase, hence they will interfere constructively in the exit medium^40^. Consequently, the first Kerker condition is met, zero backward scattering occurs, and most of electromagnetic energy can transmit through the device. Thus the forward transmittance is as high as 0.96 at *λ*_B_ = 1250 nm.

Last but not least, by comparing Fig. 4(a2) with Fig. 4(b2), one can find that the incident light at *λ*_A_ = 1000 nm and that at *λ*_B_ = 1250 nm excite ***p***_**x**_ with the same directions (both are from left to right), while their ***m***_**y**_ directions are opposite (one is outwards, the other is inwards). This also proves that the second and first Kerker conditions are respectively satisfied at the two wavelengths, and destructive interference makes light at *λ*_A_ = 1000 nm have a low transmittance of 0.02, while constructive interference enables the transmittance at *λ*_B_ = 1250 nm up to 0.96. For other wavelengths, they neither satisfy the first Kerker nor the second Kerker condition, partial interference occurs, and their transmittances are bigger than that at *λ*_A_ = 1000 nm but smaller than that at *λ*_B_ = 1250 nm. To some degree, we can say that near-field electromagnetic coupling effect, together with the first or the second Kerker conditions, determines the operation bandwidth of our proposed AT device.

The above discussions are for forward incidence, now we consider the backward incident light. Because the filling factor of the lower grating is larger (*f*_2_ = *l*/*d* = 0.67), most of the backward incident light is reflected by the lower grating. In addition, SPPs can’t be excited by the lower grating, so the backward transmittance is much lower than the forward one.

Based on the above discussion, we conclude that near-field coupling via the interaction of electric- and magnetic-dipole moments decides the transmission bandwidth of the device, enhances forward transmission at the operation wavelength.

**Figure 5:**
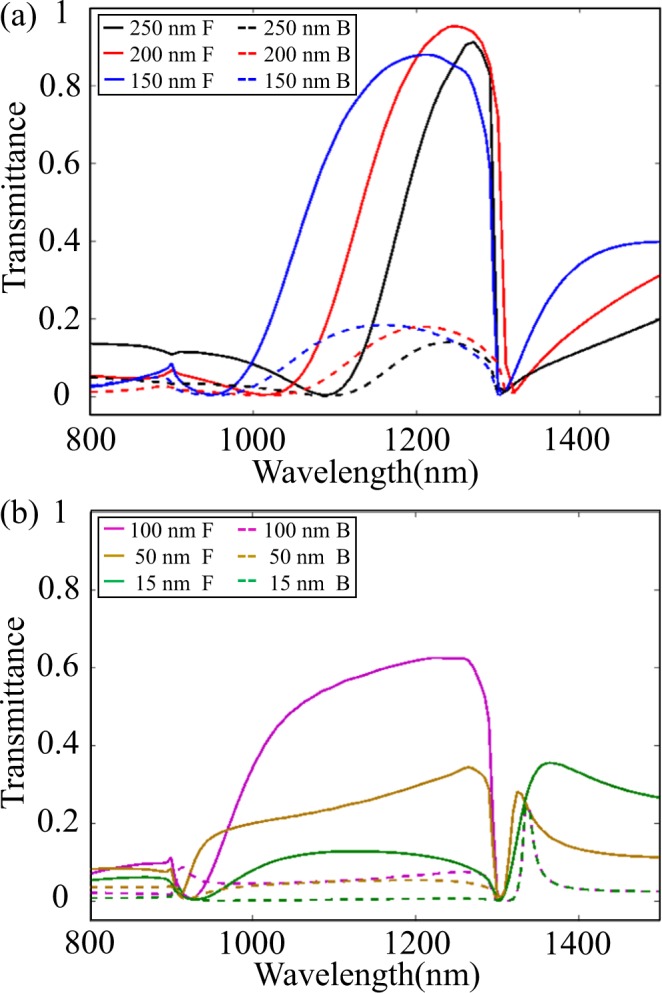
Forward and backward transmission spectra for (**a**) *t*_s_ = 250 nm (black line), *t*_s_ = 200 nm (red line) and *t*_s_ = 150 nm (blue line), (**b**) *t*_s_ = 100 nm (purple line), *t*_s_ = 50 nm (yellow line) and *t*_s_ = 15 nm (green line). “F” and “B” in the legend represent “Forward” and “Backward”, respectively.

As the spacer thickness *t*_s_ plays a critical role in vertical coupling effect, we in Fig. 5(a,b) present the forward and backward transmission spectra for various *t*_s_ values. As can be seen, when *t*_s_ = 200 nm, the forward incident light has a bigger transmittance (see the red solid line in Fig. 5(a)). When *t*_s_ is reduced, the inherent radiation loss of the grating increases because the near-field coupling between the gratings becomes stronger^41–43^, resulting in a larger spectral width. As *t*_s_ is further decreased to a certain value, *t*_s_ < 100 nm (see Fig. 5(b)), a strong and sharp Fano resonance peak appears near 1330 nm, which is worth further investigating.

### Lateral coupling

**Figure 6:**
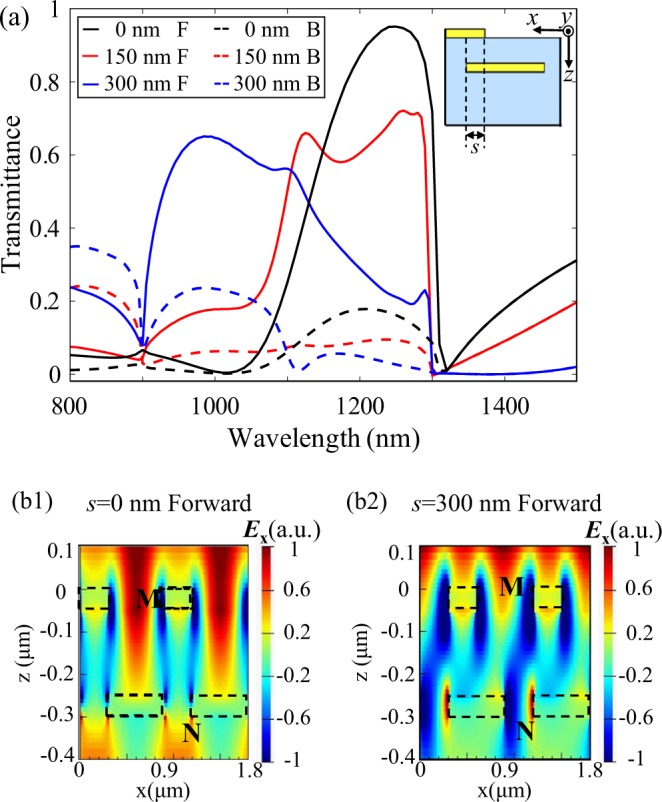
(**a**) Forward and backward transmission spectra for *s* = 0 nm (black line), *s* = 150 nm (red line), *s* = 300 nm (blue line). The inset shows the lateral misalignment *s*. *E*_x_ electric field distribution for (b1) *s* = 0 nm at *λ* = 1250 nm and (b2) *s* = 300 nm at *λ* = 1250 nm. The dotted rectangles represent the location of the upper and lower gratings. “F” and “B” in the legend represent “Forward” and “Backward”, respectively.

In the above, we focus on the vertical coupling in the complementary gratings, which depends on the spacer layer thickness *t*_s_. Now we turn to discuss the lateral coupling by setting a lateral misalignment *s* between the upper and lower gratings. The dielectric thickness *t*_s_ is fixed at 200 nm to maintain strong vertical coupling effect. Simulated forward and backward transmittance spectra for varying *s* are shown in Fig. 6(a).

When *s* increases from 0 nm to 300 nm, transmittance spectra go down and broaden. Moreover, double-peak and even multiple-peak phenomena appear when *s* = 150 nm and *s* = 300 nm. This is because when varying *s*, the effective refractive index of the bilayer gratings and the relative phase of light transmitted through the subwavelength grooves in the two gratings will also change^44–46^, as a result, resonance wavelength and transmittance spectra will change, too. Besides, the varying *s* also changes the strength of coupling effect, and then splitting of transmission peak takes place, which may lead to double or even multiple peaks and broaden transmission spectrum^47,48^.

To explore the mechanism behind this, we plot the *E*_x_ electric field distribution under *x*-polarized light forward illumination at wavelength 1250 nm for lateral misalignment *s* = 0 and *s* = 300 nm in Fig. 6(b1,b2), respectively. When *s* = 0 nm, the electric field within the subwavelength groove of the upper metallic grating (marked by position M in Fig. 6(b1)) has positive value (shown by red color in the color map), while that within the subwavelength groove of the lower metallic grating (marked by position N in Fig. 6(b1)) is also positive (represented by yellow color in the color map), indicating they are in phase, and constructive interference happens, thus enhancing forward transmission. While for the lateral misalignment *s* = 300 nm at the same wavelength, the electric field within the groove of the upper metallic grating (marked by position M in Fig. 6(b2)) has positive sign (represented by red color in the color map), while that within the groove of the lower metallic grating (marked by position N in Fig. 6(b2)) has negative sign (indicated by blue color in the color map), reflecting they are out of phase, destructive interference occurs and hence forward transmission is weaken. This implies that near-field electromagnetic coupling efficiency through subwavelength grooves and thus the AT behaviors are also strongly dependent on lateral misalignment *s*.

In a word, vertical coupling and lateral coupling both play important roles in AT performance, including forward transmittance, operation bandwidth and contrast ratio. Different spacer layer thickness *t*_s_ or lateral misalignment *s* will change the relative phase of light transmitted through the subwavelength grooves in the two gratings, modulate the interference effect of the electric- and magnetic-dipole moments, thus controlling the performance of the coupled complementary grating-based AT device. In addition, for our proposed device, the spacer thickness (*t*_*s*_ = 200 nm) is far less than the operation wavelength, therefore vertical coupling plays a more dominant role than the lateral one.

It is worth mentioning that though we do not address the fabrication of our proposed device, the fabrication process of such bilayer complementary structures can refer to ref.^36^, the two gratings can be fabricated by e-beam lithography, Ag deposition and lift-off process, and the SiO_2_ spacer layer is obtained by low pressure chemical vapor deposition.

## Discussion

To sum up, we propose and demonstrate a strong direction-selective asymmetric transmission (AT) device based on coupled complementary gratings. It consists of an upper metallic grating, a lower metallic grating which is the Babinet complementary structure of the upper one, and a dielectric spacer layer between them. Different from previous work, the two gratings are geometrically complementary to each other, and they are in asymmetric dielectric environment enabling the unidirectional excitation of SPPs and thus asymmetric optical transmission. More importantly, near-field coupling effect via the interaction of electric- and magnetic-dipole moments, which inherently exists in the coupled complementary structure, is also employed to realize high-performance AT with large forward transmittance (0.96 at the wavelength of 1250 nm), bigger 3-dB bandwidth (~174 nm). Moreover, the effects of vertical and lateral couplings for varying spacer layer thickness *t*_s_ and lateral misalignment *s* are also investigated to unearth the AT mechanism. We believe our work can help people gain a better understanding of the near-filed coupling effect in coupled complementary structures, expand their application fields, and also provide an alternate way to realize high-performance AT devices.

## Methods

Throughout the paper, the numerical simulations are performed by commercial software Lumerical FDTD Solutions. Period boundary conditions along the *x*- and *y*-directions and perfectly matched layer condition along the *z*-direction are applied. The reflective index of SiO_2_ is set as *n*_d_ = 1.5, and the dependence of silver (Ag) permittivity on optical wavelength *λ* is taken from the experimental data by Johnson and Christy^49^: ε_Ag_ = 4.0–54*λ*^2^ + i*λ*(0.38 + 0.71*λ*_2_).
